# Daily physical activity as determined by age, body mass and energy balance

**DOI:** 10.1007/s00421-015-3135-7

**Published:** 2015-02-25

**Authors:** Klaas R. Westerterp

**Affiliations:** Department of Human Biology, Maastricht University Medical Centre, PO Box 616, 6200 MD Maastricht, The Netherlands

**Keywords:** Daily energy expenditure, Activity energy expenditure, Body movement, Body composition, Obesity, Doubly labelled water, Accelerometry

## Abstract

**Aim:**

Insight into the determinants of physical activity, including age, body mass and energy balance, facilitates the design of intervention studies with body mass and energy balance as determinants of health and optimal performance.

**Methods:**

An analysis of physical activity energy expenditure in relation to age and body mass and in relation to energy balance, where activity energy expenditure is derived from daily energy expenditure as measured with doubly labelled water and body movement is measured with accelerometers, was conducted in healthy subjects under daily living conditions over intervals of one or more weeks.

**Results:**

Activity energy expenditure as a fraction of daily energy expenditure is highest in adults at the reproductive age. Then, activity energy expenditure is a function of fat-free mass. Excess body mass as fat does not affect daily activity energy expenditure, but body movement decreases with increasing fatness. Overweight and obesity possibly affect daily physical activity energy expenditure through endurance. Physical activity is affected by energy availability; a negative energy balance induces a reduction of activity expenditure.

**Conclusion:**

Optimal performance and health require prevention of excess body fat and maintenance of energy balance, where energy balance determines physical activity rather than physical activity affecting energy balance.

## Introduction

The energy cost of physical activity, as determined by body movement, is the most variable component of daily energy expenditure (Starling 2002). Habitual physical activity shows large variations from day to day due to day-to-day variations in activity behaviour of a subject. Additionally, activity behaviour can be affected by exercise training, for instance by compensatory activities (Melanson et al. 2013). Longer-term variations in physical activity within subjects and differences in activity patterns between subjects are affected by body mass. Body mass determines the energy costs of performing a physical task and therefore the question is whether body mass is a determinant of the activity pattern of a subject. Similarly, energy availability is a determinant of physical performance and therefore energy balance may be a potential determinant of the activity pattern as well. Underfeeding has been shown to have an unfavourable effect on free-living physical activity (Martin et al. 2011). Finally, activity behaviour changes as a function of development, during growth from childhood to adulthood, and with the general functional decline with subsequent ageing.

Insight into the effects of age, body mass and energy balance on daily physical activity facilitates the design of intervention studies where body mass and energy balance are determinants of health and optimal performance. To approach these research questions, analyses of daily physical activity in relation to age, body mass and energy balance are performed for studies where activity energy expenditure is derived from doubly labelled water-assessed daily energy expenditure (Speakman 1997) and body movement is measured with accelerometers (Westerterp 2009).

The doubly labelled water method is considered the gold standard for measuring daily energy expenditure under field conditions (Shephard and Aoyagi 2012). It allows measuring energy expenditure in unrestrained individuals over a time interval of 1–4 weeks. Accelerometers provide additional information on body movement with regard to the amount and intensity over much shorter intervals, usually minutes, to assess activity patterns throughout days and weeks.

## Methods of measuring daily physical activity

**Fig. 1:**
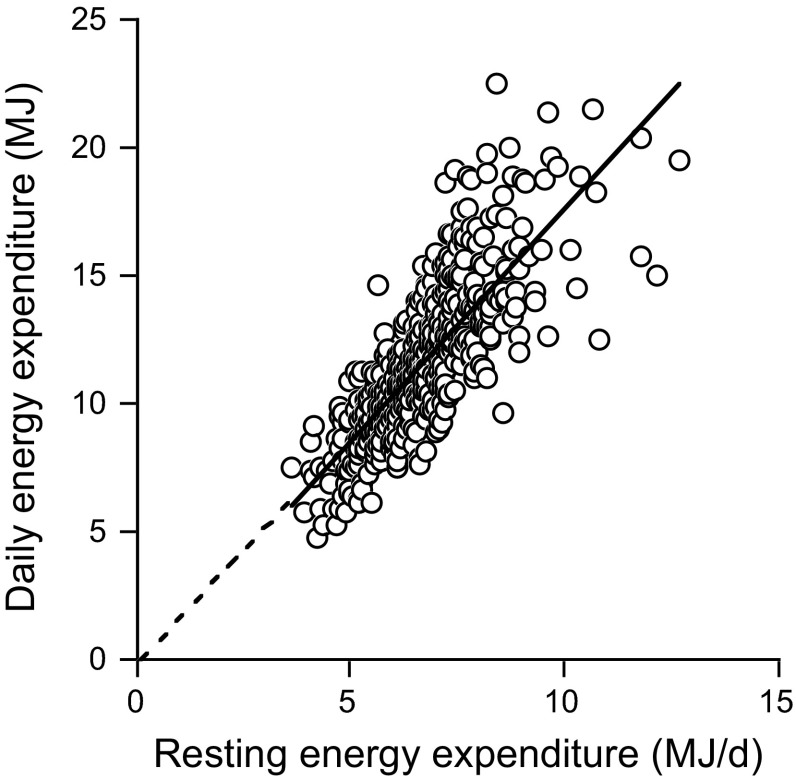
Daily energy expenditure plotted as a function of resting energy expenditure for 529 adult subjects with the extrapolated linear regression line (Speakman and Westerterp 2010)

The indicated method for the measurement of activity energy expenditure is the doubly labelled water method for the measurement of daily energy expenditure in combination with a measurement of resting energy expenditure. Daily energy expenditure consists of three components, resting energy expenditure, the energy cost of food processing or diet-induced thermogenesis and the energy cost of physical activity. Resting energy expenditure is usually the largest component of daily energy expenditure and is mainly a function of body composition (Starling 2002). Diet-induced thermogenesis, depending on what and how much one eats, amounts to a fixed fraction of about 10 % of daily energy expenditure for a mixed diet consumed according to energy requirement (Westerterp 2004). Activity energy expenditure is calculated as 0.9 × daily energy expenditure − resting energy expenditure, where diet-induced energy expenditure is estimated as 10 % of daily energy expenditure. Alternatively, the physical activity level is calculated as daily energy expenditure divided by resting energy expenditure: physical activity level = daily energy expenditure/resting energy expenditure (FAO/WHO/UNU 2004). Daily energy expenditure divided by resting energy expenditure adjusts for subject characteristics, resulting in a dimensionless figure allowing for comparison of activity levels between subjects differing in body size and body composition. Analysis of measurements of 529 adult subjects shows that the intercept of the regression of daily energy expenditure on resting energy expenditure is not different from zero (Fig. 1), confirming the utility of physical activity level for comparisons of physical activity (Speakman and Westerterp 2010).

The doubly labelled water method is the gold standard for the validation of field methods of assessing physical activity. The indicated method for the assessment of body movement in daily life is a doubly labelled water-validated accelerometer (Westerterp 2014). Accelerometers provide information on the total amount, the frequency, the intensity and the duration of physical activity. Accelerometer-assessed body movement allows further insight into these aspects of physical activity as determined by age, body mass and energy balance. Data included in the analyses are from studies in healthy subjects observed under daily living conditions over intervals of one or more weeks.

## Effects of age and body mass on daily physical activity

Age and body mass are determinants of variation in activity-induced energy expenditure. Physical activity level is analysed in relation to growth and age by comparing young children and adults. Physical activity level in adults is analysed in relation to being underweight or overweight. Subsequently, physical activity level is combined with data on accelerometer-assessed body movement, illustrating interaction between physical activity and body mass.

**Fig. 2:**
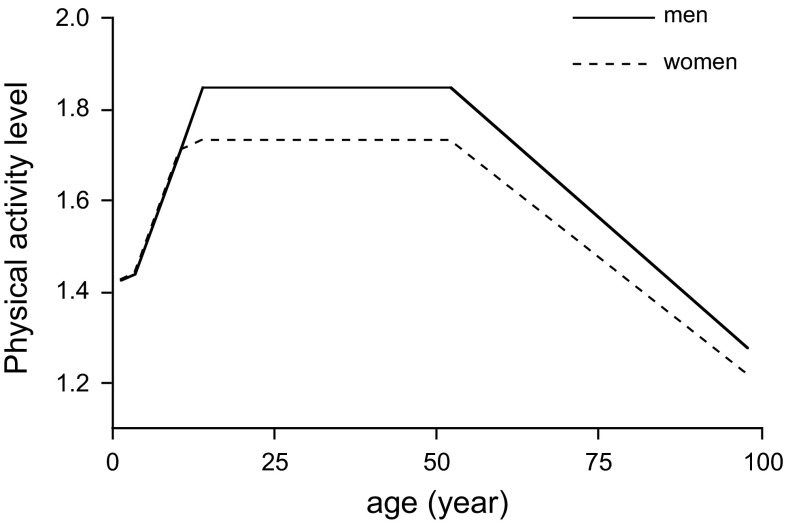
Physical activity level for women and men with increasing age (FAO/WHO/UNU 2004; Speakman and Westerterp 2010)

Body mass increases from 3 to 4 kg at birth to adult value of 60–70 kg. Doubly labelled water data on physical activity level in 1- to 18-year-olds were analysed by an FAO/WHO/UNU expert group (FAO/WHO/UNU 2004). Data from adult humans between 18 and 96 years of age were recently compiled as well (Speakman and Westerterp 2010). The physical activity level increased from an average of 1.4 at age 1 to 1.75 at age 15 (Fig. 2). On average, the physical activity level between ages of 18 and 50–55 years averaged 1.75 in women and 1.84 in men. Above age 50–55 years, physical activity level was generally lower and declined to a value around 1.3 for both sexes in subjects aged 90–100 years. Thus, it seems that physical activity level is highest when adult body mass and muscle mass are reached. The decline after age 50 might be associated with the age-related fat-free mass loss and fat mass gain, where at the same body mass one gets relatively fatter and less muscular. Similarly, men with a lower body fat percentage have slightly higher physical activity level values than women with a higher body fat percentage. The data as compiled in Fig. 2 present the development of physical activity level with age for healthy subjects observed under daily living conditions, with a focus on growth and ageing. The development of physical activity level shows that physical activity is the highest in adults at the reproductive age.

**Fig. 3:**
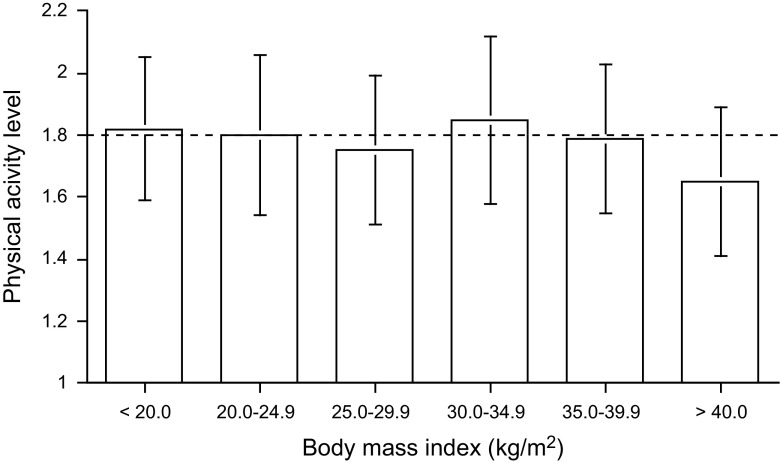
Physical activity level and body mass index (Westerterp and Speakman 2008)

Most data for analysing physical activity level in relation to body mass are available for adults. Physical activity level can be compared between weight categories by adjusting weight for height with the body mass index (BMI), where body mass is divided by height squared: BMI = body mass/height^2^ (kg/m^2^). Analysis of 319 measurements of physical activity level in adults aged 18–64 years showed that physical activity level was quite similar at different levels of BMI (Prentice et al. 1996). A more recent analysis of 366 measurements of physical activity level in adults aged 18–50 years gave similar results (Westerterp and Speakman 2008). The average physical activity level is around 1.80 for all body mass index categories except the very highest (Fig. 3). The average physical activity level value for subjects with a body mass index higher than 40 kg/m^2^, i.e., for subjects with morbid obesity, was 1.65 ± 0.24.

Daily energy expenditure increases with body mass as a function of fat-free mass (Webb 1981; Schoeller and Fjeld 1991). Similarity of physical activity level values for subjects in different weight categories implies that activity energy expenditure is a function of fat-free mass as well. Thus, activity energy expenditure/kg body mass is negatively related to body fat percentage with consequences for body movement. In a comparative study in obese subjects and non-obese controls with the same fat-free mass-adjusted activity energy expenditure, accelerometer-assessed body movement was lower in obese subjects than in non-obese controls (Ekelund et al. 2002). Fatter subjects generally move less, because daily energy expenditure is not higher in proportion to the higher fat percentage and to the higher cost for weight-bearing activities.

**Fig. 4:**
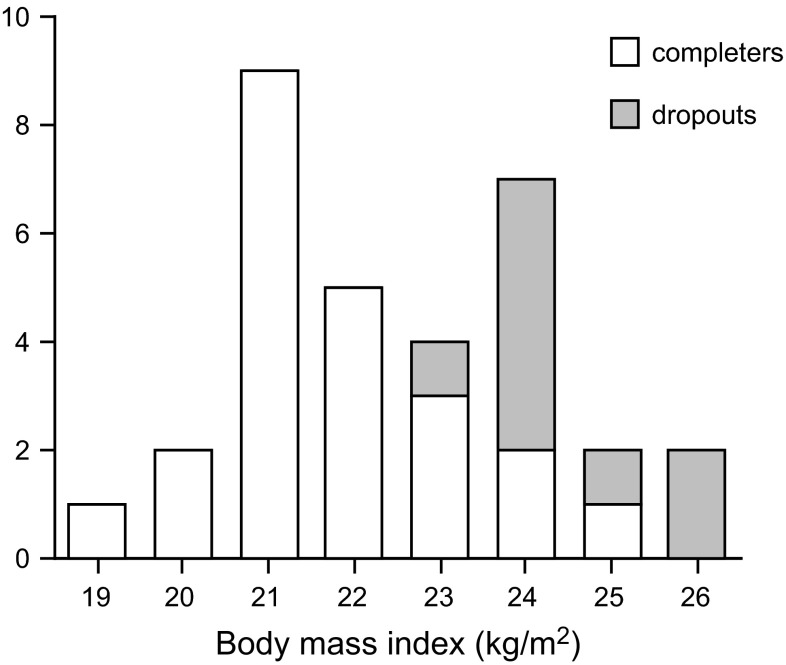
Frequency distribution of body mass index of 23 subjects who completed training and 9 who were dropouts to run a half-marathon (Westerterp et al. 1992)

For the same physical activity level, lean subjects can move more than fat subjects. Obese subjects walk slower than lean subjects (Kim et al. 2013). Obese subjects have increased muscular strength, but reduced muscular endurance. The 6-min walking distance decreases nearly linearly with increasing BMI (Pataky et al. 2014). Physical performance seems to be already limited in subjects at the higher end of the normal range of BMI. In a study to prepare subjects to run a half-marathon, 9 out of 32 subjects withdrew on being unable to keep up with the training programme (Westerterp et al. 1992). All dropouts had a BMI between 23 and 26 kg/m^2^, i.e., above the group average, but not yet indicating that they were overweight (Fig. 4). A lower BMI facilitates physical capacity combined with the advantage of a low body mass during weight-bearing activities. Extremely thin subjects, especially subjects with anorexia nervosa, tend to be excessively physically active (Kron et al. 1978; Falke et al. 1985). However, there seems to be a lower limit for physical performance and BMI as well. At a BMI below 17 kg/m^2^, subjects showed relatively low duration of moderate and high-intensity activities indicating a declining physical capacity (Bouten et al. 1996).

## Energy balance and physical activity

There is day-to-day variation in energy balance through variation in food intake and physical activity. On a daily basis, food intake and energy expenditure do not correlate. However, the correlation between intake and expenditure improves considerably on a weekly basis (Edholm et al. 1955). Generally, days with a high physical activity are followed by an increased intake with a lag time of 3–6 days (Champagne et al. 2013), resulting in energy balance on a weekly basis. Active subjects seem to compensate quicker for an activity-induced energy deficit than inactive individuals (Rocha et al. 2013). The effect of energy balance on physical activity can be derived from studies on overeating and energy restriction over intervals of one or more weeks.

Several studies estimated the effect of overfeeding on physical activity level (Westerterp 2010). Subjects were overfed with 20–100 % over 2–10 weeks. There does not seem to be an effect of a positive energy balance, as induced by overfeeding, on physical activity. Only massive overfeeding, doubling intake over 9 weeks, affected physical activity (Pasquet et al. 1992). Then, physical activity level went down from 1.87 ± 0.12 to 1.45 ± 0.09 (*p* < 0.001), activity energy expenditure decreased by 30 % and accelerometer-assessed body movement decreased by 40 %.

**Fig. 5:**
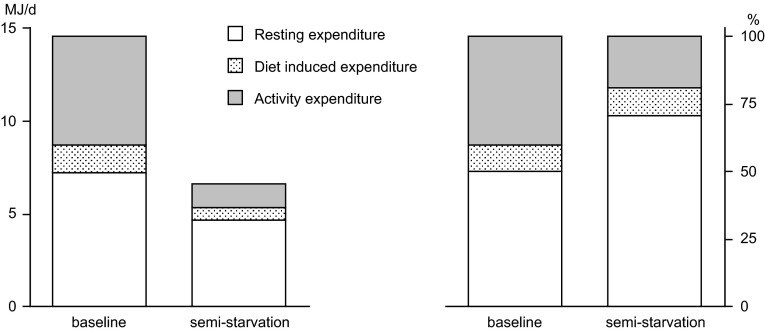
The components of daily energy expenditure in MJ/d (*left*) and in per cent of daily energy expenditure (*right*), at baseline and after 24 weeks of semi-starvation (Keys et al. 1950)

Studies on energy restriction, generally in overweight and obese subjects, show little or no effect of underfeeding on physical activity level (Westerterp 2013). However, a classical underfeeding study in normal weight subjects, the so-called Minnesota Experiment, showed a reduction of physical activity during long-term semi-starvation (Keys et al. 1950). The weight maintenance diet of young men was reduced to 50 % during 24 weeks. At the end of the 24-week interval, subjects reached a new energy balance where energy expenditure equalled energy intake. The largest reduction of energy expenditure could be attributed to decreased activity energy expenditure, mainly through a reduction of body movement (Fig. 5). A recent study on weight loss in overweight and obese subjects showed a weight loss-induced reduction in physical activity, recovering during weight maintenance (Camps et al. 2013a). The physical activity level and accelerometer-assessed body movement decreased in a period of energy restriction and returned to baseline levels when energy balance was reached again during weight maintenance. Physical activity is affected by energy availability, where a negative energy balance induces a reduction of activity expenditure. Thus, optimal performance requires maintenance of energy balance.

## Exercise training and energy balance

It has been suggested that modern inactive lifestyles are the predominant factor in the increasing prevalence of overweight and obesity (Prentice and Jebb 1995). They suggest, the physical activity level and thus energy needs should have declined faster than energy intake as encouraged by the increasing availability of highly palatable foods. However, analysis of doubly labelled water-assessed physical activity level for trends over time showed that activity energy expenditure did not decline over the same period that obesity rates increased, and daily energy expenditure of modern man is in line with energy expenditure in wild mammals (Westerterp and Speakman 2008). Additionally, a recent study showed that daily energy expenditure was similar for subjects with Western lifestyles and traditionally living Hazda hunter–gatherers in a savannah-woodland environment in Northern Tanzania (Pontzer et al. 2012). Therefore, it is unlikely that decreased expenditure has fuelled the obesity epidemic. Experimental studies on the effect of exercise on energy balance as reviewed below indicate that exercise hardly contributes to a diet-induced negative energy balance.

Weight loss achieved with exercise training appears to be modest and is typically less than 3 % of the initial body mass (Jakicic 2009). There is resistance to exercise-induced weight loss through compensatory behavioural adaptations like reduced non-training activity and increased energy intake (Melanson et al. 2013). Long-term studies on exercise training show that the less-than-predicted weight loss mainly results from a compensatory increase in energy intake (Thomas et al. 2012). Regular exercise in previously sedentary subjects does not result in a negative compensatory reduction in nonprescribed physical activity, regardless of the type of exercise (Turner et al. 2010; Rangan et al. 2011). Exercise-induced reductions of nonprescribed physical activity is restricted to subjects at a higher age (Westerterp and Plasqui 2004), where initial physical activity level is already low as well (Fig. 2). Despite moderate exercise-induced weight loss, there are favourable exercise-induced changes in body composition, especially in fatter subjects. In the study preparing subjects to run a half-marathon, subjects with the highest BMI dropped out (Fig. 4). In the completers, women lost on average 2 kg body fat and gained 2 kg fat-free mass. The 12 men completing the training lost on average 4 kg body fat and gained 3 kg fat-free mass, where the loss of body fat was positively correlated with the initial percentage body fat (Westerterp et al. 1992). Aerobic training seems to be the optimal exercise mode for reducing body fat and resistance training for increasing fat-free mass. Thus, resistance training might even result in an increase in body mass (Willes et al. 2012). Higher exercise doses do not necessarily imply a larger change in body mass or body composition. In overweight and obese subjects, a moderate dose of exercise induced a markedly greater negative energy balance than a higher dose (Rosenkilde et al. 2012).

Early reviews on the effect of exercise in combination with energy restriction on energy balance showed an exercise programme in addition to an energy-restricted diet does not result in additional weight loss. Diet-only and diet-plus-exercise groups did not differ with respect to the amount of body mass lost or fat mass lost (Ballor and Poehlman 1994). Exercise provides some conservation of fat-free mass during weight loss by dieting, probably by maintaining glycogen and water (Garrow and Summerbell 1995). In a study randomizing overweight subjects to diet only, diet and endurance training or diet and resistance training until BMI was less than 25 kg/m^2^, all groups had similar weight loss and length of time to reach target BMI (Del Corral et al. 2009). Diet adherence was a function of weight loss and adversely affected by severity of the negative energy balance. Another study showed non-compliance to prescribed physical activity masking the effect of physical activity to further increase a diet-induced negative energy balance (DeLany et al. 2014). Additionally, weight loss induces metabolic adaptations including a decline in resting energy expenditure below the predicted values, based on the new body composition reached after weight loss (Camps et al. 2013b). Adding resistance training to an energy-restricted diet did not alter resting energy expenditure differently from a diet-only group (St-Onge et al. 2013). Even vigorous exercise did not prevent the weight loss-induced decline in resting energy expenditure despite relative preservation of fat-free mass (Johannsen et al. 2012). In the long term, both diet-only and diet-plus-exercise interventions are associated with weight regain. A meta-analysis of seven studies lasting 2 years or longer showed a weight loss averaging 1.6 kg after a combination of energy restriction and increased physical activity, 1.1 kg greater than for diet only (Wu et al. 2009). It seems difficult to successfully lose weight after becoming overweight.

## Physical activity and long-term maintenance of energy balance

The body mass of adults is regulated at a constant level. One of the earliest longitudinal studies providing information on the constancy of body mass is the Framingham Study. A group of 5209 adults, 30–59 years of age and living in the town of Framingham at the start of the study in 1948, underwent every 2 years a medical examination including measurement of body mass for at least 20 years unless prevented by illness or death. Nearly no one retained a constant body mass, but most people gained or lost between 5 and 10 kg over any part of the 20-year period in adult life (James 1985). A weight change of 1 kg/year represents an energy balance within 30 MJ/year (Westerterp 1995). Knowing that an adult has a daily energy turnover of 8–12 MJ under normal living conditions (Black et al. 1996), i.e., a mean energy turnover of 10 MJ/day or 3650 MJ/year, the discrepancy is less than 1 %. In the last decades, the prevalence of being overweight and obesity has increased worldwide. Analysis of doubly labelled water measurements of daily energy expenditure as available over the last decades suggests that physical activity level did not decline over the time obesity rates went up (Westerterp and Speakman 2008). The relation between daily energy expenditure and body mass suggests that increase in energy intake has driven the increase in body mass (Swinburn et al. 2009).

A neutral or slightly positive energy balance results in the maintenance of fat-free mass during midlife. As stated in the section on body mass and physical activity, physical activity level is highest when adult body mass and muscle mass are reached. The decline in physical activity level after age 50 does not seem to cause the age-related decline in fat-free mass loss and fat mass gain, whereas at the same body mass one gets relatively fatter and less muscular. Ageing is associated with the loss of fat-free mass, even in weight-stable subjects remaining physically active (Hughes et al. 2002). There is no relation between age-adjusted physical activity level and fat-free mass (Speakman and Westerterp 2010), and physical activity does not seem to alter the trajectory of fat-free mass change in later life (Manini et al. 2009). Functional decline at later age seems to be inevitable.

A physically active lifestyle has consequences for the maintenance of energy balance as reflected in the fat store of the body. A physically active lifestyle inevitably results in a larger decrease of physical activity level at later age than a sedentary lifestyle. The change to a lower physical activity level does not induce an equivalent reduction in energy intake. Varying physical activity level from 1.8 to 1.4 over 7 days was not associated with a change of energy intake and there was no tendency for energy intake to drop as the sedentariness progressed (Stubbs et al. 2004). Thus, the reduction of physical activity level resulted in a positive energy balance, most of which was stored as fat. Adults observed at an age of 27 ± 5 years with a physical activity level of 1.81 ± 0.16 showed a significant reduction of the physical activity level to 1.75 ± 0.11 when observed 11 ± 4 years later. There was a significant association between the change in physical activity level and the change in body fat, where a high initial activity level was predictive for a higher fat gain (Westerterp and Plasqui 2009).

## Discussion and conclusions

Physical activity level is highest when adult body mass and muscle mass is reached. The decline after age 50 might be associated with the age-related fat-free mass loss and fat mass gain, whereas at the same body mass one gets relatively fatter and less muscular. Fatter subjects generally move less because activity energy expenditure is not higher in proportion to the higher fat mass and thus the higher costs for weight-bearing activities. A lower fat mass, and thus a relatively high fat-free mass, facilitates physical capacity with the advantage of a low body mass during weight-bearing activities. A positive energy balance does not seem to affect physical activity-induced energy expenditure, while a negative energy balance induces a reduction in body movement as well as in activity energy expenditure. Thus, optimal performance requires maintenance of energy balance. Energy balance is primarily a function of energy intake. Exercise programs generally do not result in weight loss because of a compensatory increase of intake. Eating less is the most effective method for preventing weight gain, despite the decrease in physical activity at a negative energy balance.

The low physical activity level in young children can be explained by growth. In young children, resting energy expenditure is relatively high while muscle mass and other body components are growing. Young children sleep most of the day, and as they grow older they sleep less and spend more time on physical activities, resulting in higher physical activity level. Between age 15 and 20, adult body mass is reached and physical activity level reaches an adult value as well. The increase of physical activity level during growth was explained by the increase in body mass, because in children and adolescents there was no relation between weight-adjusted activity energy expenditure (activity energy expenditure/kg) and age (Hoos et al. 2003). A low physical activity level value in young children does not necessarily imply a low body movement. A small body requires less energy to move around.

Normal growth is positively associated with physical activity level. Excess growth as body fat, resulting in overweight and obesity, is not associated with a change in physical activity level. Overweight and obese subjects generally have similar activity energy expenditure while metabolic costs are higher. Fatter children are already less moderate to vigorous physically active compared to normal weight children (Haerens et al. 2007). They perform less on exercise tests and participate less in sports activities. Overweight and obesity negatively affect gait through lower speed, shorter strides and increased step width, resulting in higher cost of walking (Peyrot et al. 2009). Obese adolescents showed an improvement of walking economy after weight loss (Peyrot et al. 2012). Overweight and obese subjects can do less at a similar activity energy expenditure, and loss of excess body fat is the indicated approach to improve activity behaviour.

Body fat gain and body fat loss are a function of energy balance, where energy balance is primarily a function of energy intake (Westerterp 2010). Eating less is the most effective method for preventing weight gain. Fatness leads to inactivity, but inactivity does not lead to fatness (Metcalf et al. 2011). There is little evidence that more physically active subjects gain less excess weight than more sedentary subjects (Cook and Schoeller 2011). Eating less is the most effective method for preventing weight gain, despite the decrease in physical activity at a negative energy balance.

In conclusion, activity energy expenditure as a fraction of daily energy expenditure is similar for lean, overweight and obese subjects. Fatter subjects generally move less, because daily energy expenditure and activity energy expenditure are a function of the fat-free mass and not higher in proportion to the higher cost for weight-bearing activities in subjects with a higher fat mass. Maintenance of physical activity and physical performance requires maintenance of energy balance, where energy balance determines physical activity rather than physical activity affecting energy balance.

## Abbreviations

BMI: Body mass index
FAO/WHO/UNU: Food and Agriculture Organization/World Health Organization/United Nations University
